# 基于增强MRI诊断并行全脑放疗的非小细胞肺癌脑转移预后分析

**DOI:** 10.3779/j.issn.1009-3419.2011.09.05

**Published:** 2011-09-20

**Authors:** 辉 刘, 琼雅 吴, 晓梅 宫, 晓东 贺, 红宇 吴, 兆瑛 盛, 道安 周

**Affiliations:** 200433 上海，同济大学附属上海市肺科医院放疗科 Department of Radiation Oncology, Shanghai Pulmonary Hospital, Tongji University School of Medicine, Shanghai 200433, China

**Keywords:** 肺肿瘤, 脑/核磁共振成像, 放疗, 预后, Lung neoplasms, Magnetic resonance imaging (MRI), Radiotherapy, Prognosis

## Abstract

**背景与目的:**

肺癌脑转移约占脑转移瘤的20%-40%。本研究旨在探讨基于增强MRI诊断并行全脑放疗的非小细胞肺癌（non-small cell lung cancer, NSCLC）脑转移的预后因素。

**方法:**

回顾性分析2007年4月-2008年10月241例NSCLC脑转移并接受全脑放疗的病例资料，采用*Kaplan-Meier*法计算生存率，*Log-rank*法进行单因素分析，*Cox*回归分析进行多因素分析。

**结果:**

中位随访时间为19.1个月，全组中位生存时间为8.7个月。影响NSCLC脑转移生存的单因素包括女性、KPS（karnofsky performance score） > 70分、脑转移无症状、胸内病变控制、化疗3周期以上及合并靶向治疗。多因素分析显示性别、随访截止时胸内病变控制状态、靶向治疗是影响NSCLC脑转移生存的独立预后因素。

**结论:**

对于基于增强MRI诊断并行全脑放疗的NSCLC脑转移患者，性别、胸内病变控制、靶向治疗是影响生存的独立预后因素。

## Abstract (en)

**Background and objective:**

Brain metastases are a common complication of lung cancer, occurring in 20%-40% of patients. The aim of this study is to explore prognostic factors in non-small cell lung cancer (NSCLC) in patients with brain metastases diagnosed by constrast-enhanced MRI after whole brain radiotherapy.

**Methods:**

TA retrospective review of clinical data from 241 NSCLC patients with brain metastases received whole brain radiotherapy from April 2007 to October 2008 was performed. A number of potential factors that might affect prognosis after irradiation were evaluated. The significance of prognostic variables in the survival resulted from univariate analysis by *Kaplan-Meier* combining with *Log-rank* test, and the multivariate analysis was obtained by *Cox* regression model.

**Results:**

Median follow-up time for the survivors was 19.1 months. For all patients, the median survival time (MST) was 8.7 months. By univariate analysis, female patients with KPS > 70, no symptom when diagnosed with brain metastases, tumor controlled in the chest, and received more than 3 cycles of chemotherapy and combined target therapy were the important factors for overall survival. By multivariate analysis, female, tumors controlled in the chest, and combined target therapy were independent prognostic factors for NSCLC patients with brain metastases. Tumor controlled in the chest was the most important independent prognostic factor.

**Conclusion:**

Gender, local tumor controlled, and combined target therapy significantly influenced NSCLC brain metastases diagnosed by constrast-enchanced MRI survival after whole brain radiotherapy.

脑转移瘤中原发部位以肺癌最为多见，约占脑转移瘤的20%-40%。增强MRI（magnetic resonance imaging）扫描是脑转移的最佳检查方法，其诊断脑转移较增强CT（computed tomography）更敏感^[1]^。因此有学者认为基于CT诊断和基于MRI诊断的脑转移在预后和治疗原则上可能存在不同^[2]^。迄今对于肺癌脑转移的治疗多为姑息治疗，治疗方法的合理选用要根据患者的自身条件，肿瘤的位置、大小及数目，肿瘤的临床分期和病理类型作出综合判断。本研究通过分析基于增强MRI诊断的NSCLC脑转移的生存情况，探讨其预后因素，为临床治疗决策提供依据。

## 1 材料与方法

### 1.1 入组分析的条件

① 完整病例资料；②经组织病理学或细胞学证实为NSCLC；③增强MRI证实为脑转移；④在同济大学附属上海市肺科医院接受全脑放射治疗。

### 1.2 一般资料

见表 1。2007年4月-2008年10月在同济大学附属上海市肺科医院接受全脑放疗的NSCLC患者241例。男女比例为1.5:1，中位年龄为57岁（29岁-83岁）。

**1:** 基于MRI诊断的241例NSCLC患者的一般资料和单因素分析结果 Characteristics and univariate analysis of overall survival in 241 NSCLC patients with brain metastases diagnosed by constrast-enhanced MRI

Characteristics		*n*	MST (month)	1-year survival rate	2-year survival rate	*P*
Gender	Male	144	6.5	29.9	13.1	0.011
	Female	97	11.4	45.4	18.5	
Age (yr)	> 65	64	6.0	26.6	13.6	0.067
	≤65	177	9.5	40.1	16.0	
KPS	> 70	179	9.8	40.2	18.1	0.007
	≤70	62	6.8	25.8	7.3	
Pathology	Adenocarcinoma	165	9.3	37.6	15.8	0.218
	Non-adenocarcinoma	76	6.0	34.2	13.9	
Symptoms	Yes	17	5.6	17.6	5.9	0.022
	No	224	8.7	37.9	16.0	
Tumor size	≥2 cm	30	4.9	26.7	8.3	0.058
	< 2 cm	211	8.8	37.9	16.4	
Numbers	1-3	143	8.7	35.7	18.7	0.387
	> 3	98	8.5	37.8	10.0	
CEA	≥40	71	9.2	35.2	16.5	0.499
	< 40	170	8.1	37.1	14.6	
Tumor status in thoracic	Progression	182	6.8	25.8	6.0	< 0.001
	Stable	59	16.2	69.5	47.8	
Tumor tatus intracalvarium	Progression	181	9.1	38.5	19.5	0.150
	Stable	60	9.1	38.5	19.5	
Thoracic radiotherapy	Yes	24	11.5	41.7	12.5	0.667
	No	217	8.4	35.9	15.7	
Chemotherapy	≥3 cycles	140	10.1	44.3	23.2	< 0.001
	< 3 cycles	101	6.2	25.7	3.0	
Target therapy	Yes	67	13.6	61.2	32.1	< 0.001
	No	174	6.7	27.0	8.8	
MST: median survival time; KPS: karnofsky performance score; CEA: carcinoembryonic antigen. NSCLC: non-small cell lung cancer; MRI: magnetic resonance imaging.						

### 1.3 脑转移诊断及放疗

全组病例均经增强MRI诊断为脑转移，MRI常规扫描行横断T1WI、T2WI及水抑制成像，增强扫描分别行横断、矢状、冠状T1WI像扫描，层厚均为3 mm，造影剂为GdDTPA。241例患者中3个以上的多发脑转移灶者最常见，占全组的53.5%（129/241）；单发转移灶者次之，占31.5%（76/241）。可评价转移部位共239处，额叶转移最常见，占64.4%（154/239）；顶叶次之，占38.5%（92/239）。全脑放疗中位剂量为30 Gy（9 Gy-30 Gy），因多发转移且转移灶 < 2 cm，96.2%（232/241）的患者接受30 Gy/10次的剂量照射后未局部加量，9例患者全脑放疗后接受局部常规/X-加量放疗。

### 1.4 其它治疗情况

全组患者病程中均接受中位3周期全身化疗（1周期-9周期）。67例接受靶向治疗，占27.8%（67/241）。接受胸部放疗者占10%（24/241），中位剂量为60 Gy。3例患者曾接受脑转移瘤切除术。22例在肺癌初治时接受肺叶切除术，1例接受全肺切除术。

### 1.5 随访及疗效评价

治疗后每2个月复查1次，以门诊和电话随访为主。主要观察指标为总生存时间。

### 1.6 统计学方法

应用SPSS 13.0进行统计处理。采用*Kaplan-Meier*法计算生存率，*Log-rank*法行差异分析。多因素分析采用*Cox*回归分析（Enter法）。*P* < 0.05为差异有统计学意义。

## 2 结果

### 2.1 生存情况

截至2009年9月17日，患者中位随访时间19.1个月（15.0-28.2）。全组生存38例，死亡203例，中位生存时间（median survival time, MST）为8.7个月（0.7-31.1），1年生存率为36.5%，2年生存率为5.3%。

### 2.2 影响NSCLC脑转移生存的单因素分析

见表 1。女性、KPS（karnofsky performance score） > 70分、脑转移无症状、胸内病变控制、化疗3周期以上及合并靶向治疗提示脑转移后生存时间长。男性和女性MST分别为11.4个月和6.5个月（*P*=0.011）；KPS > 70分和KPS≤70分的MST分别为9.8个月和6.8个月（*P*=0.007）。年龄 > 65岁、脑转移瘤≥2 cm有影响NSCLC脑转移预后的趋势，但与对照组相比MST差异无统计学意义（*P*=0.067, *P*=0.058）。颅内肿瘤控制情况对生存无影响（*P*=0.15）。

### 2.3 影响NSCLC脑转移生存的多因素分析

分析发现KPS与化疗周期数明显相关，KPS > 70分者接受3周期以上化疗的占64.8%，而≤70分者占35.2%（*P*=0.001），因此化疗周期数未纳入多因素分析。*Cox*回归分析（Enter法）显示，性别、随访截止时胸内病变控制状态及靶向治疗是影响NSCLC脑转移生存的独立预后因素（表 2），胸内病变控制状态是较强的独立预后因素（*P* < 0.001, RR=2.971, 95%CI: 1.987-4.440）。

**2:** 基于MRI诊断的NSCLC 241例多因素分析 Multivariate analysis of overall survival in 241 NSCLC patients with brain metastases diagnosed by constrast-enhanced MRI

Variable	B	SE	Wald	Sig	Exp (B) 95%CI
Gender (male *vs* female)	-0.357	0.157	5.674	0.017	0.700 (0.522-0.939)
Age (≤65 *vs* > 65)	0.277	0.17	2.661	0.103	1.319 (0.946-1.839)
KPS (> 70 *vs* ≤70)	0.287	0.161	3.192	0.074	1.332 (0.973-1.826)
Brain metastases tumor size (≥2 cm *vs* < 2 cm)	-0.218	0.222	0.964	0.326	0.804 (0.520-1.243)
Thoracic tumor status (stable *vs* progression)	1.809	0.205	28.177	< 0.001	2.971 (1.987-4.440)
Target therapy (no *vs* yes)	-0.603	0.176	11.75	0.001	0.547 (0.387-0.772)

## 3 讨论

20世纪50年代肾上腺皮质激素被发现可改善颅内转移患者的症状，并使脑转移患者的MST由1个月延长至2个月。20世纪70年代全脑放射治疗成为脑转移的标准治疗方案，MST提高至大约4个月-6个月，同时提高了生存质量。文献^[3, 4]^报道NSCLC脑转移患者MST为3.2个月-7个月，1年生存率约为10%-15%。本研究全组241例NSCLC脑转移患者全脑放疗后MST为8.7个月，1年和2年生存率分别为37.1%和15.5%，疗效好于文献报道，可能与本研究中脑转移瘤多为小病灶有关， < 2 cm的转移瘤占87.6%。另外基于增强MRI诊断的现代诊断技术对多发的小转移灶和无症状的脑转移检出率提高^[2]^，更多的小病变、无症状的脑转移患者接受全脑放疗及合并靶向治疗都可能是本研究中患者生存时间延长的因素。

影响NSCLC脑转移的预后因素包括KPS、年龄、颅外转移状态和转移瘤数目^[5]^。本研究显示KPS > 70分、胸内病变稳定、女性及合并靶向治疗是影响NSCLC脑转移生存的独立预后因素。NSCLC脑转移中女性患者生存时间长于男性，具体机理尚不清楚，可能与女性内在的生物学行为及某些蛋白质的高表达有关，如甲状旁腺相关蛋白（parathyroid hormone-related protein, PTHrP）、ERCC1（excision repair cross complementry 1）、Her2（human epidermal growth factor receptor 2）及RXRβ等，这些物质具有潜在地抑制内皮细胞增殖、迁移及肿瘤生长的作用。对于脑转移的NSCLC患者，颅外无其它转移或胸内病变控制者预后好^[6, 7]^。本研究也显示胸内病变控制状态是较强的独立预后因素（*P* < 0.001, RR=2.971），脑转移时胸内病变进展者MST仅为3.7个月，在随访截止时胸内病变稳定者较胸内病变进展者MST长9.4个月。但本研究并未观察到接受胸部放疗者有生存获益，这可能与接受放疗的病例数少有关，如何结合全身化疗和局部放疗来提高胸内病变控制还需要临床实践进一步的验证。

表皮生长因子受体酪氨酸激酶抑制剂（epidermal growth factor receptor-tyrosine kinase inhibitors, EGFR-TKIs）包括吉非替尼和厄洛替尼，是治疗晚期NSCLC有效的靶向治疗药物，可以透过血脑屏障，对脑转移有治疗作用^[8-13]^。2011年吴等^[13]^报道了48例无症状的脑转移患者接受厄洛替尼治疗的总体有效率为56.3%。本研究显示化疗3周期以上及合并靶向治疗患者脑转移后生存时间长。但患者的一般状况与化疗的周期数明显相关，一般状况较好的KPS > 70分的患者接受3周期以上化疗的占65.8%，而70分的患者占35.2%（*P*=0.001），因此考虑化疗周期数并不能真正反映患者的预后，而接受靶向治疗才是NSCLC患者独立的预后因素。NSCLC脑转移全脑放疗序贯联合吉非替尼或厄洛替尼者，MST明显长于未接受靶向治疗者（13.6个月*vs* 6.8个月, *P* < 0.001）。但从EGFR-TKIs中获益的NSCLC患者仍有26%出现孤立的中枢神经系统失败^[14]^。全脑放疗如何联合靶向治疗药物控制脑转移病变、提高患者生存时间和生活质量是今后治疗的方向。

## References

[1] Ouyang H, Zhou CW, Zhang HM (2004). MRI features of brain metastases of lung cancer. Zhonghua Zhong Liu Za Zhi.

[2] Seute T, Leffers P, ten Velde GP (2008). Detection of brain metastases from small cell lung cancer: consequences of changing imaging techniques (CT versus MRI). Cancer.

[3] Oh Y, Taylor S, Bekele BN (2009). Number of metastatic sites is a strong predictor of survival in patients with nonsmall cell lung cancer with or without brain metastases. Cancer.

[4] Videtic GM, Reddy CA, Chao ST (2009). Gender, race, and survival: a study in non-small-cell lung cancer brain metastases patients utilizing the radiation therapy oncology group recursive partitioning analysis classification. Int J Radiat Oncol Biol Phys.

[5] Sperduto PW, Chao ST, Sneed PK (2010). Diagnosis-specific prognostic factors, indexes, and treatment outcomes for patients with newly diagnosed brain metastases: a multi-institutional analysis of 4, 259 patients. Int J Radiat Oncol Biol Phys.

[6] Flannery TW, Suntharalingam M, Regine WF (2008). Long-term survival in patients with synchronous, solitary brain metastasis from non-small-cell lung cancer treated with radiosurgery. Int J Radiat Oncol Biol Phys.

[7] Sanchez de Cos J, Sojo Gonzalez MA, Montero MV (2009). Non-small cell lung cancer and silent brain metastasis. Survival and prognostic factors. Lung Cancer.

[8] Kim JE, Lee DH, Choi Y (2009). Epidermal growth factor receptor tyrosine kinase inhibitors as a first-line therapy for never-smokers with adenocarcinoma of the lung having asymptomatic synchronous brain metastasis. Lung Cancer.

[9] Ma S, Xu Y, Deng Q (2009). Treatment of brain metastasis from non-small cell lung cancer with whole brain radiotherapy and Gefitinib in a Chinese population. Lung Cancer.

[10] Wang Y, Wang Y, Wang B (2006). Primary result of the efficacy and tolerance of gefitinib in advanced non-small cell lung cancer patients with brain metastasis. Zhongguo Fei Ai Za Zhi.

[11] Wu C, Li LY, Wang MZ (2007). Gefitinib in the treatment of advanced non-small cell lung cancer with brain metastasis. Zhonghua Zhong Liu Za Zhi.

[12] Shukuya T, Takahashi T, Naito T (2011). Continuous EGFR-TKI administration following radiotherapy for non-small cell lung cancer patients with isolated CNS failure. Lung Cancer.

[b13] 13Wu YL, Zhou C, Cheng Y, *et al*. A phase Ⅱ study (CTONG0803) of erlotinib as second-line treatment in advanced non-small cell lung cancer (NSCLC) patients with asymptomatic brain metastases (BM) after first-line chemotherapy (CT). ASCO, 2011: poster 7605.

[14] Lee YJ, Choi HJ, Kim SK (2010). Frequent central nervous system failure after clinical benefit with epidermal growth factor receptor tyrosine kinase inhibitors in Korean patients with non small-cell lung cancer. Cancer.

